# Effect of probiotics combined with Ulinastatin and Somatostatin in the treatment of severe acute pancreatitis

**DOI:** 10.12669/pjms.40.8.9744

**Published:** 2024-09

**Authors:** Hehe Dou, Yue Kan, Zhipeng Xu, Zhenjie Wang, Chuanming Zheng

**Affiliations:** 1 Hehe Dou, Department of Emergency Surgery, Bengbu Medical College, First Affiliated Hospital, Bengbu, Anhui Province 233000, P.R. China; 2 Yue Kan, Department of Pain, The Second Affiliated Hospital of Guangxi Medical University, Nanning, Guangxi Province 530000, P.R. China; 3 Zhipeng Xu, Department of Emergency Surgery, Bengbu Medical College, First Affiliated Hospital, Bengbu, Anhui Province 233000, P.R. China; 4 Zhenjie Wang, Department of Emergency Surgery, Bengbu Medical College, First Affiliated Hospital, Bengbu, Anhui Province 233000, P.R. China; 5 Chuanming Zheng, Department of Emergency Surgery, Bengbu Medical College, First Affiliated Hospital, Bengbu, Anhui Province 233000, P.R. China

**Keywords:** Probiotics, Ulinastatin, Somatostatin, Severe Acute Pancreatitis

## Abstract

**Objective:**

To evaluate the clinical effect of probiotics combined with Ulinastatin and Somatostatin in the treatment of severe acute pancreatitis.

**Methods:**

A retrospective study was conducted on 160 patients with severe acute pancreatitis treated in the First Affiliated Hospital of Bengbu Medical College from July 2021 to June 2023. There were 78 patients received Ulinastatin and Somatostatin treatment (Control group), and 82 patients received probiotics in addition to Ulinastatin and Somatostatin treatment (Observation group). The treatment effect and the time required to alleviate clinical symptoms were compared between the two groups. Serum levels of inflammatory factors, intestinal mucosal indexes and the incidence of adverse reactions before and after treatment were analyzed.

**Results:**

The total efficacy of the Observation group (95.12%) was higher than that of the Control group (85.90%) (*P*<0.05). Combined probiotic/Ulinastatin + Somatostatin treatment was associated with shorter time to remission of the clinical symptoms (*P*<0.05). After the treatment, serum levels of inflammatory factors in the two groups were decreased, and was significantly lower in the Observation group compared to the Control group (*P*<0.05). Similarly, post-treatment serum levels of intestinal mucosal indexes in the two groups were lower than before the treatment, and significantly lower in the Observation group (*P*<0.05). There was no significant difference in the incidence of adverse reactions between the groups (*P*>0.05).

**Conclusions:**

A combined regimen of probiotics, Ulinastatin and Somatostatin is safe and can more effectively relieve clinical symptoms in patients with severe acute pancreatitis, reduce levels of inflammatory factors, lower intestinal mucosal damage and improve the overall treatment effect compared to Ulinastatin and Somatostatin regimen alone.

## INTRODUCTION

Acute pancreatitis is a common disease with an estimated incidence of approximately 13-45 per 100,000 population, and the incidence continues to increase globally over time.^1^,^2^ Acute pancreatitis involves acute inflammation of the pancreatic parenchyma and surrounding tissues, and is most commonly caused by gallstones, alcohol use, and hypertriglyceridemia.^3^ Pancreatitis is manifested as persistent epigastric pain,^3^,^4^ and if not treated in a timely and effective manner, may progress to pancreatic necrosis that is associated with multiple organ dysfunction and increased mortality rates.^4^,^5^

Ulinastatin and Somatostatin are commonly used in the treatment of acute pancreatitis.^6^,^7^ Somatostatin is a protease inhibitor that suppresses the production of digestive enzymes, reduces the secretion of gastrin and insulin, induces apoptosis of pancreatic acinar cells, and prevents gastrointestinal injury caused by trypsin.^7^ Ulinastatin is a glycoprotein, which can reduce the absorption of toxin, inhibit the production of tumor necrosis factor, improve the stability of vascular endothelial cells, reduce the activity of trypsin, and reduce myocarditis and myocardial injury.^6^-^8^

Recently, the application value of probiotics in severe acute pancreatitis became a focus of research.^9^,^10^ Studies show that probiotics can inhibit the growth of pathogenic bacteria in the intestinal tract, protect the intestinal mucosal barrier function, enhance intestinal motility, improve intestinal immune function, and, subsequently, alleviate pancreatic injury.^9^-^11^ However, few studies have investigated the combined effects of probiotics and Ulinastatin and Somatostatin. In the current study, we aimed to explore the therapeutic effect of probiotics in combination with Ulinastatin and Somatostatin in patients with severe acute pancreatitis, and to provide reference for the clinical treatment of the disease.

## METHODS

A retrospective study was conducted on 160 patients (94 males and 66 females) with severe acute pancreatitis treated in the First Affiliated Hospital of Bengbu Medical College from July 2021 to June 2023. A total of 78 patients were treated with Ulinastatin and Somatostatin and were selected as the Control group, while 82 patients who were treated with a combination of probiotics, Ulinastatin and Somatostatin were set as the Observation group.

### Ethical Approval:

This study was approved by the ethics committee of the First Affiliated Hospital of Bengbu Medical College (No.: 2023-419; Date: November 1, 2023). Informed consent was not required due to the retrospective nature of the study. The study was carried out in accordance with the declaration of Helsinki. Patient personal data were anonymized and stored in an encrypted computer.

### Inclusion criteria:

- Patients diagnosed as severe acute pancreatitis.^12^,^13^
- Age ≥ 18 years old.
- Complete clinical data.

### Exclusion criteria:

- Bile duct obstruction and gallstone.
- A history of biliary surgery.
- Other organ infections.
- Benign and malignant tumors.
- Lactation and pregnancy.
- Acute intestinal obstruction and peptic ulcer.
- Allergic constitution.

### Treatment procedures:

All patients were given a standard symptomatic support including antibiotics, rehydration, gastrointestinal decompression, fasting and water deprivation. Ulinastatin (Guangdong techpool Biochemical Pharmaceutical Co., Ltd.) was administered as intravenous infusion of 100000U Ulinastatin +250 ml glucose solution (5%) three times a day, for a total of 10 days. Somatostatin (Kunming Longjin Pharmaceutical Co., Ltd.) was administered by micro pump with 3mg Somatostatin+50 ml normal saline at the rate of 4 ml/hour for 10 days. For patients in the combined group, Probiotic Bifidobacterium Tetravaccine tablets (Hangzhou Longda Xinke biopharmaceutical Co., Ltd.) were injected from the stomach tube in suspension, 5g/time, three times/day for 10 days.

### Observed indicator:

- Therapeutic effect was classified as markedly effective, effective, or invalid. Markedly effective: yellow staining of mucosa, skin and sclera disappeared, and the bilirubin level returned to normal; Effective: yellow staining of mucosa, skin and sclera disappeared, bilirubin levels decreased but did not return to normal; Invalid: the yellow staining of mucosa, skin and sclera has not disappeared, and the bilirubin levels have not returned to normal. Total effective rate = (markedly effective + effective)/total number of cases × 100%.
- Time for clinical symptom relief, including the recovery time of bowel sounds, disappearance time of vomiting and nausea, disappearance time of abdominal distension and abdominal pain, and the recovery time of blood amylase.
- Inflammatory factors, including tumor necrosis factor -α (TNF-α), white blood cell (WBC), interleukin-6 (IL-6), C-reactive protein (CRP) levels, were detected in the fasting venous blood by enzyme-linked immunosorbent assay. The corresponding kits were purchased from Shanghai enzyme linked Biotechnology Co., Ltd.
- Intestinal mucosal index, including D-lactic acid (DLA), diamine oxidase (DAO), was measured in the fasting venous blood by enzyme-linked immunosorbent assay using corresponding kits from Shanghai enzyme linked Biotechnology Co., Ltd.
- Incidence of adverse reactions: rash, diarrhea, vomiting and nausea.

### Statistical analysis:

All data were analyzed by SPSS 25.0 software (IBM Corp, Armonk, NY, USA). The normality of the data was evaluated by Shapiro-Wilk test. The data of normal distribution were expressed as mean ± standard deviation. Independent sample t-test was used for inter-group comparison, and paired t-test was used for intra-group comparison. The data of non-normal distribution were expressed by median and interquartile interval. Mann Whitney U test was used for comparison between the groups, and Wilcoxon signed rank test was used for comparison within the groups. The counting data were expressed as number of cases and percentage, and compared using Chi-square test. *P*-value less than 0.05 was considered statistically significant. All reported *P* values were bilateral.

## RESULTS

A total of 160 patients who met the eligibility criteria were included in the analysis. Age of the patients ranged from 26 to 59 years, with an average age of 43.58 ± 10.97 years. There was no significant difference in the basic information between the two groups of patients (*P*<0.05) (Table-I).

**Table-I:** Comparison of Basic Information between Two Groups.

Group	Gender (Male/Female)	Age (Year)	BMI (kg/m²)	Causes of illness		

				Alcoholic	Biliary	Hyperlipidemia
Observation group (n=82)	51/31	42.89±11.36	23.30±2.96	31 (37.81)	35 (42.68)	16 (19.51)
Control group (n=78)	43/35	44.31±10.57	23.72±3.02	27 (34.62)	42 (53.85)	9 (11.53)
*χ^2^*/*t*	0.824	-0.816	-0.889	2.774		
P	0.364	0.416	0.375	0.250		

The total efficacy of the Observation group (95.12%) was higher than that of the Control group (85.90%) (*P*<0.05) (Table-II). The recovery time for bowel sounds, vomiting and nausea, abdominal distension and pain, and blood amylase were significantly shorter in the Observation group compared to the Control group (*P*<0.05) (Table-III).

**Table-II:** Comparison of clinical efficacy between two groups.

Group	n	Markedly effective	Effective	Invalid	Total effective rate (%)
Observation group	82	51 (62.20)	27 (32.93)	4 (4.88)	78 (95.12)
Control group	78	39 (50.00)	28 (35.90)	11 (14.10)	67 (85.90)
*χ^2^*					4.004
*P*					0.045

**Table-III:** Comparison of clinical symptom relief time between two groups (day).

Group	n	Bowel sounds	Vomiting and nausea	Abdominal distension and pain	Blood amylase
Observation group	82	1.51±0.42	3.19±1.05	4.43±1.14	5.31±1.10
Control group	78	2.12±0.60	4.93±1.26	5.69±1.35	6.78±1.79
*t*		7.480	9.508	6.390	6.292
*P*		<0.001	<0.001	<0.001	<0.001

Before the treatment, there was no significant difference in serum levels of TNF-α, WBC, IL-6, CRP between the two groups (*P*>0.05). After the treatment, the serum levels of TNF-α, WBC, IL-6, and CRP in both groups decreased, and were significantly lower in the Observation group than in the Control group (*P*<0.05) (Table-IV).

**Table-IV:** Comparison of two groups of inflammatory factors.

Time	Group	n	TNF-α (ng/L)	WBC (×10^9^/L)	IL-6 (ng/L)	CRP (mg/L)
Before treatment	Observation group	82	18.82±3.06	19.11±3.38	17.79±3.14	24.34±3.86
	Control group	78	19.20±2.86	18.95±3.51	18.10±3.06	24.17±3.79
	*t*		0.811	0.294	0.632	0.281
	*P*		0.419	0.769	0.528	0.779
After treatment	Observation group	82	6.40±1.98^a^	4.94±1.23^a^	6.81±2.51^a^	5.39±1.72^a^
	Control group	78	10.14±2.51^a^	7.37±1.59^a^	9.63±2.60^a^	7.79±1.93^a^
	*t*		10.491	10.843	6.980	8.313
	*P*		<0.001	<0.001	<0.001	<0.001

***Note:*** Compared with the same group before the treatment,

a P<0.05.

Before the treatment, serum levels of DLA and DAO were comparable in the two groups (*P*>0.05). After the treatment, the levels of serum DLA and DAO in both groups decreased, and were significantly lower in the Observation group compared to the Control group (*P*<0.05) (Table-V). There was no significant difference in the incidence of adverse reactions between the Observation and the Control group (4.88% and 2.56%, respectively) (*P*> 0.05) (Table-VI).

**Table-V:** Comparison of intestinal mucosal indicators between two groups.

Time	Group	n	DLA (mg/L)	DAO (U/L)
Before treatment	Observation group	82	13.05±2.08	18.12±3.53
	Control group	78	12.95±2.24	17.99±3.79
	*t*		0.293	0.225
	*P*		0.770	0.823
After treatment	Observation group	82	4.38±1.11^a^	4.96±1.56^a^
	Control group	78	6.87±1.52^a^	7.59±2.35^a^
	*t*		11.875	8.378
	*P*		<0.001	<0.001

***Note:*** Compared with the same group before the treatment,

a *P*<0.05.

**Table-VI:** Comparison of adverse reactions between two groups.

Group	n	Rash	Diarrhea	Vomiting and nausea	Total incidence rate (%)
Observation group	82	1 (1.22)	1 (1.22)	2 (2.44)	4 (4.88)
Control group	78	0 (0.00)	1 (1.28)	1 (1.28)	2 (2.56)
*χ^2^(Continuity correction)*					0.125
*P*					0.723

## DISCUSSION

The results of this study showed that in patients with severe acute pancreatitis, the regimen that combines probiotics with Ulinastatin and Somatostatin is associated with significantly shorter symptom relief time, and higher total effective rate of the treatment, with no significant increase in the incidence of adverse reactions.

Ulinastatin and Somatostatin are commonly used in the treatment of pancreatitis. Ulinastatin can reduce the activity of trypsin and other trypsin-related substances, improve the stability of lysosomal membrane, reduce the production of myocardial inhibitory factors and lysosomal enzymes, and regulate pancreatic microcirculation.^7^,^8^ Somatostatin is a neurohormone that can inhibit endocrine, reduce the production of glucagon, insulin and pancreatic enzyme, reduce pancreatic secretion, and promote the recovery of pancreatic function.^7^,^8^,^14^ Horváth et al.^14^ in a meta-analysis of nine randomized controlled trials involving 1037 patients, showed that Ulinastatin combined with Somatostatin in the treatment of acute pancreatitis can significantly relieve symptoms and shorten hospitalization time. Studies showed that Ulinastatin combined with Somatostatin can prevent pancreatic proenzyme activation and enzyme secretion, and prevent further aggravation of local pancreatic lesions.^6^,^7^

However, in our study, the total efficacy of pancreatitis patients treated with Somatostatin and Ulinastatin alone was only 85.90%, and was increased to 95.12% after adding probiotics. In recent years, studies have confirmed the clinical value of probiotics in pancreatitis. Wan et al.^15^ showed that the hospitalization time and abdominal pain relief time of patients with mild acute pancreatitis were shortened after taking probiotics as adjuvant therapy on the basis of conventional drugs. Werawatganon et al.^16^ used the mouse model of acute pancreatitis to show that probiotics can alleviate the degree of pancreatitis reaction and help restore intestinal integrity. The study also showed that probiotics can regulate intestinal flora, reduce endotoxin and bacterial absorption, enhance immune function, protect intestinal mucosa, and promote the rehabilitation of pancreatitis. Mores et al.^17^ also confirmed that probiotics play a positive role in improving the therapeutic effect and prognosis of pancreatitis. The study also showed that supplementing exogenous probiotics could increase the number of Enterococcus faecalis, Bifidobacterium and Lactobacillus acidophilus in the intestine, improve intestinal mucosal function, enhance gastrointestinal motility, and promote the absorption of nutrients. A study by Liu^18^ also confirmed that adding probiotics as adjuvant treatment to the enteral nutrition can improve the overall treatment effect and promote the early recovery of patients with pancreatitis. The above studies are all consistent with our results, further confirming that probiotics have important adjuvant value in the treatment of pancreatitis.

Inflammatory factors play an important role in the pathogenesis and progression of pancreatitis. The abnormal production of cytokines and inflammatory mediators such as TNF-α, WBC, IL-6, CRP in pancreatitis is caused by excessive activation of nuclear transcription factor NF -κβ pathway and toll pathway that trigger the cascade amplification reaction, potentially leading to multiple organ dysfunction and failure.^19^,^20^ Xu et al.^21^ showed that in patients with acute pancreatitis, Ulinastatin combined with Somatostatin can reduce inflammatory reaction and enhance immune function. In our study, the post-treatment levels of TNF-α, WBC, IL-6 and CRP in patients who received probiotics as an adjuvant treatment, were lower than those in the Control group. We may speculate that this effect is due to ability if probiotics to regulate the production of anti-inflammatory and pro-inflammatory factors, improve intestinal immune function, alleviate the degree of inflammatory reaction in the body, and restore the immune balance, which is consistent with the observations of Xu et al.^21^ and Chen et al.^22^

Diamine oxidase (DAO), and D-lactic acid (DLA) levels can be used to evaluate the therapeutic effect and prognosis of pancreatitis.^23^ DAO is a type of intracellular enzyme in gastrointestinal mucosa that can be used to evaluate the degree of gastrointestinal mucosal injury. Similarly, increased levels of DLA, a bacterial metabolite in the intestine, are indicative of the damaged intestinal barrier function.^24^ The results of this study showed that DAO and DLA levels in the Observation group were lower than those in the Control group after the treatment, further confirming that probiotics combined with Ulinastatin and Somatostatin was feasible and effective in the treatment of pancreatitis. Our results are consistent with the observation of Gu et al.^25^ Moreover, Patel et al.^26^ showed that probiotics can promote the intestinal immune defense function through defensins, improve the immune state of the body, stimulate the production of biological enzymes and vitamins, enhance the activity of natural killer cells and phagocytes, accelerate the generation of antibodies, eliminate oxygen free radicals in the intestinal tract, and reduce intestinal injury. It is, therefore, plausible that exogenous probiotics can upregulate the expression of intestinal epithelial intercellular linker, promote the recovery of intestinal mucosa, improve epithelial integrity, improve mucosal permeability, restore barrier function, prevent pathogenic bacteria from entering the blood through intestinal mucosa, and prevent intestinal infection.^25^-^27^

### Limitations:

Firstly, this is a retrospective study, which means there is a risk of selection bias. Secondly, we did not account for possible external factors during hospitalization, including non-medical factors that may affect our results. Finally, there was no long-term follow-up data, and the long-term effect of treatment needs to be verified. Further higher quality research is needed to verify our conclusions.

## CONCLUSION

Probiotics combined with Ulinastatin and Somatostatin in the treatment of severe acute pancreatitis are safe and can effectively relieve the clinical symptoms of patients, reduce the level of inflammatory factors, reduce intestinal mucosal damage, improve the overall treatment effect.

### Authors’ contributions:

**HD** conceived and designed the study.

**YK, ZX, ZW and CZ** collected the data and performed the analysis.

**HD** was involved in the writing of the manuscript and is responsible for the integrity of the study.

All authors have read and approved the final manuscript.
